# The Interaction between Plants and Bacteria in the Remediation of Petroleum Hydrocarbons: An Environmental Perspective

**DOI:** 10.3389/fmicb.2016.01836

**Published:** 2016-11-21

**Authors:** Panagiotis Gkorezis, Matteo Daghio, Andrea Franzetti, Jonathan D. Van Hamme, Wouter Sillen, Jaco Vangronsveld

**Affiliations:** ^1^Environmental Biology, Centre for Environmental Sciences, Hasselt UniversityDiepenbeek, Belgium; ^2^Department of Environmental Sciences, University of Milano-BicoccaMilano, Italy; ^3^Department of Biological Sciences, Thompson Rivers University, KamloopsBC, Canada

**Keywords:** phytoremediation, bioremediation, remediation, petroleum hydrocarbons, plant–bacteria assisted remediation

## Abstract

Widespread pollution of terrestrial ecosystems with petroleum hydrocarbons (PHCs) has generated a need for remediation and, given that many PHCs are biodegradable, bio- and phyto-remediation are often viable approaches for active and passive remediation. This review focuses on phytoremediation with particular interest on the interactions between and use of plant-associated bacteria to restore PHC polluted sites. Plant-associated bacteria include endophytic, phyllospheric, and rhizospheric bacteria, and cooperation between these bacteria and their host plants allows for greater plant survivability and treatment outcomes in contaminated sites. Bacterially driven PHC bioremediation is attributed to the presence of diverse suites of metabolic genes for aliphatic and aromatic hydrocarbons, along with a broader suite of physiological properties including biosurfactant production, biofilm formation, chemotaxis to hydrocarbons, and flexibility in cell-surface hydrophobicity. In soils impacted by PHC contamination, microbial bioremediation generally relies on the addition of high-energy electron acceptors (e.g., oxygen) and fertilization to supply limiting nutrients (e.g., nitrogen, phosphorous, potassium) in the face of excess PHC carbon. As an alternative, the addition of plants can greatly improve bioremediation rates and outcomes as plants provide microbial habitats, improve soil porosity (thereby increasing mass transfer of substrates and electron acceptors), and exchange limiting nutrients with their microbial counterparts. In return, plant-associated microorganisms improve plant growth by reducing soil toxicity through contaminant removal, producing plant growth promoting metabolites, liberating sequestered plant nutrients from soil, fixing nitrogen, and more generally establishing the foundations of soil nutrient cycling. In a practical and applied sense, the collective action of plants and their associated microorganisms is advantageous for remediation of PHC contaminated soil in terms of overall cost and success rates for *in situ* implementation in a diversity of environments. Mechanistically, there remain biological unknowns that present challenges for applying bio- and phyto-remediation technologies without having a deep prior understanding of individual target sites. In this review, evidence from traditional and modern omics technologies is discussed to provide a framework for plant–microbe interactions during PHC remediation. The potential for integrating multiple molecular and computational techniques to evaluate linkages between microbial communities, plant communities and ecosystem processes is explored with an eye on improving phytoremediation of PHC contaminated sites.

## Introduction

Petroleum hydrocarbons (PHCs) are organic compounds comprised of carbon and hydrogen atoms arranged in varying structural configurations with physical and chemical characteristics that vary over orders of magnitude; they are broadly classified in two categories namely, gasoline range organics (GROs) and diesel range organics (DROs). GROs include mono-aromatic hydrocarbons such as benzene, toluene, ethylbenzene, and xylenes (BTEX), and short chain alkanes (C6–C10) with low boiling points (60–170°C) such as isopentane, 2,3-dimethyl butane, *n*-butane, and pentane. DROs include longer chain alkanes (C10–C40) and hydrophobic chemicals such as polycyclic aromatic hydrocarbons (PAH) (Kamath et al., 2004). The industrialization of modern societies and the increasing demand for energy generation to heat our domestic and working areas, to fuel our transportation networks as well as to power fabricating processes has resulted in the extensive exploitation of PHCs, which are the most widespread class of organic contaminants worldwide (Brassington et al., 2007).

Prolonged exposure to PHCs can initiate detrimental damages to the central nervous system in humans and animals, can result in respiratory system dysfunction, disrupt the endocrine system and, as a result, considerably increase the probability of lung, skin, bladder, liver, and kidney cancers (Costello, 1979; Hutcheson et al., 1996; Boffetta et al., 1997; Singh et al., 2004; Locksley, 2010). Hence, the need to remediate PHC contaminated environments is of great importance.

Generally, conventional physical and chemical *in situ* and *ex situ* clean-up technologies for PHC remediation involve excavation, air sparging, removal and off-site treatment in biopiles, pump and treat, incineration, slurry- and solid phase reactors, soil washing, soil vapor extraction, asphalt batching, thermal desorption, chemical oxidation, hydrolysis and photolysis (Amatya et al., 2002; Khan et al., 2004; Zhou et al., 2005; Do et al., 2009). However, experience has demonstrated that these strategies are expensive, and often only result in incomplete decomposition of the pollutants of the concern.

Thus, research over the last two decades has focused on offering remediation schemes that are moving away from the conventional ones and are mainly based on biological methods with emphasis to the convergent action of plants and their related microorganisms to remove and degrade PHCs. However, there are still numerous aspects about the mechanisms involved that remain the subject of research and debate among members of the scientific community.

This review tries to provide one more piece of information in this complicated puzzle of plant–microbe partnerships with emphasis on the remediation of PHC contaminated sites mediated by plant–bacteria associations.

## Bioremediation of Petroleum Hydrocarbons

**FIGURE 1:**
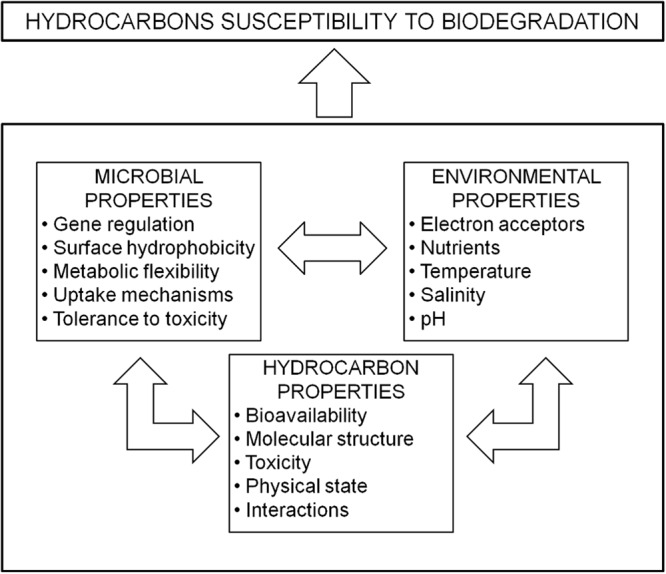
**Main factors affecting biodegradation of petroleum hydrocarbons (PHCs)**.

Bioremediation is defined as the use of biologically mediated processes to detoxify, degrade or transform pollutants to an innocuous state (Azubuike et al., 2016). Bioremediation is a useful tool for the treatment of PHC contaminated terrestrial and marine ecosystems (Atlas, 1995; Atlas and Cerniglia, 1995; Almeida et al., 2013; Xue et al., 2015; Scoma et al., 2016; Wang et al., 2016). Accession to PHC substrates while regulating toxic effects is the first hurdle that must be overcome for a microorganism to exploit these energy-rich molecules for growth and energy production. The pivotal parameters that dictate the degree of PHC “susceptibility” to biodegradation can be widely classified into three inter-related categories (**Figure 1**): (a) **microbial properties** (genetic complement, gene regulation and expression, surface hydrophobicity, metabolic diversity and flexibility, substrate uptake or adherence mechanisms, tolerance to metals and other toxic xenobiotics, chemotaxis, biofilm formation); (b) **environmental factors** (presence of terminal electron acceptors, nutrient availability, salinity, pressure, temperature, pH, water availability, and osmotic stress); and (c) **properties of the hydrocarbon substrate** (solubility, concentration, hydrophobicity, volatility, molecular mass) (Sikkema et al., 1995; Hino et al., 1997; Marquez-Rocha et al., 2001; Bressler and Gray, 2003; Martinez-Checa et al., 2007; Bordoloi and Konwar, 2009; Botalova et al., 2009; Calvo et al., 2009; Banat et al., 2010; Couling et al., 2010).

Generally, once a bacterial community begins to remove PHCs from a contaminated environment, bioavailability (here defined as “the quantity of a contaminant which is freely available to cross the cellular membrane of an organism from the surrounding medium”), and bioaccessibility [here defined as the “quantity of the contaminant which has the potential to cross an organism’s (cellular) membrane from the environment it inhabits”], determine the degree and rate at which the contaminant can be taken up by the microorganism (Semple et al., 2007; Dandie et al., 2010). Moreover, bioavailability may be assessed in two complementary ways: (i) by chemical methods (e.g., selective extraction methods), which determine the available fraction of a well-defined class of contaminants, and (ii) by biological methods, which expose organisms to contaminated media (Harmsen, 2007). Although plethora of reports supports the concept that bioremediation efficiency is normally limited by PHC bioavailability (Schwartz and Scow, 2001; Wick et al., 2001; Liste and Alexander, 2002; Shor et al., 2003; Tabak et al., 2003; Hamdi et al., 2007b), such generalizations should not be applied to all cases (Huesemann et al., 2004) given the diversity of the biological world.

In a classical experiment conducted by Rosenberg et al. (1980), the microbial adhesion to hydrocarbon (MATH) assay was established as a method to quantify microbial cell surface hydrophobicity via their attachment to hydrocarbon droplets. Other quantitative measures of cell hydrophobicity include the measurement of water contact angles (Reid et al., 1992) and zeta potentials (Busscher et al., 1995).

Microbial adhesion to hydrophobic surfaces, usually defined as the process of transferring unbound, suspended cells from the aqueous phase to an interface (pure or mixed, liquid or solid hydrocarbons in a water-immiscible phase), is one mechanism used by microorganisms to counteract the limited bioavailability of insoluble and poorly soluble PHCs (Bouchez-Naitali et al., 1999; Hermansson, 1999). The significance of adhesion in the biodegradation of aliphatic hydrocarbon non-aqueous phase liquids (NAPLs) has been reported by Volkering et al. (1997); however, adherence to PHCs does not necessarily correlate with utilization (Grimaud, 2010).

Depending on the physiology of the organism involved, microbial adhesion to hydrophobic surfaces may benefit growth on, and biodegradation of, very poorly water-soluble PHCs such as *n*-alkanes and large PAHs dissolved in a non-aqueous phase (Abbasnezhad et al., 2011). In other cases, the addition of cationic surfactants such as cetylpyridinium chloride (CPC), poly-L-lysine and chlorhexidine gluconate (CHX), or long chain alcohols such as 1-dodecanol and farnesol, may promote the growth of a hydrophilic bacterium, such as *Pseudomonas fluorescens* strain LP6a, at oil–water interfaces (Abbasnezhad et al., 2008).

Biosurfactants, either microbially derived or plant derived, can also be involved in hydrocarbon accession by regulating cell envelope hydrophobicity and, thus, the attachment and detachment to and from PHC droplets. This can be facilitated by exposing the hydrophilic or hydrophobic moieties of cell-bound biosurfactants external to the cell (Rosenberg et al., 1988). Microorganisms with degradation capabilities may also alter their cell hydrophobicity during growth on PHCs (Franzetti et al., 2008a; Tzintzun-Camacho et al., 2012).

Interestingly, it has been found that the qualitative and quantitative composition of bacterial outer surfaces are affected in a dose-dependent manner by biosurfactants such as rhamnolipids (Zhong et al., 2007; Sotirova et al., 2008), fatty acids (Chang et al., 2009), and chemical surfactants (Mohanty and Mukherji, 2012).

## Biosurfactants, Biofilms, and Chemotaxis: Role in Improving Bioremediation

Bacteria, yeast and filamentous fungi can synthesize a structurally diverse array of organic compounds with surface activity. These amphiphilic compounds generally comprise a hydrophilic acid, peptide cations or anions, mono-, di- or polysaccharides, and a hydrophobic moiety of unsaturated or saturated hydrocarbon chains, fatty acids, or lipids (Banat et al., 2010). Surface active compounds in biological systems can be broadly classified as: (a) low-molecular-weight compounds called **biosurfactants**, such as lipopeptides, glycolipids, and proteins (e.g., glycolipids such as rhamnolipids, trehalose lipids, sophorolipids, mannosylerythritol lipids, and lipopeptides such as surfactin and fungicin (Franzetti et al., 2008a, 2010; Cameotra and Singh, 2009; Nguyen and Sabatini, 2011; Banat et al., 2014; Dobler et al., 2016; Santos et al., 2016); and (b) **bioemulsifiers**, high-molecular-weight polymers of lipopolysaccharides, polysaccharides, proteins or lipoproteins (e.g., such as the lipopolysaccharide emulsan and the polysaccharide and protein complex alasan) (Neu, 1996; Uzoigwe et al., 2015). Biosurfactants reduce surface and interfacial tensions, while bioemulsifiers stabilize oil-in-water emulsions and have less capacity to lower surface tension than biosurfactants (Smyth et al., 2010a,b).

Microbial surfactants can promote bacterial growth on PHCs by increasing the surface area between oil and water through emulsification, and by increasing pseudosolubility through partitioning into micelles (Volkering et al., 1997). In certain cases, this results in an increase in contaminant bioavailability to degrading microorganisms. Recent reviews provide paradigms of successful biosurfactant applications in bioremediation processes (Mulligan, 2009; Pacwa-Plociniczak et al., 2011; Lawniczak et al., 2013). For example, production of lipopeptides by *Bacillus circulans* (Das et al., 2008), as well as lipopeptides and protein-starch-lipids by two strains of *Pseudomonas aeruginosa* (Bordoloi and Konwar, 2009) have been shown to enhance PAH biodegradation.

Relatively recently, a comparative study between Triton X-100 and the commercial rhamnolipid JBR-515 (Jeneil Biosurfactant Company, USA), was conducted to explore the factors affecting the process of surfactant enhanced biodegradation of model NAPLs by a naphthalene degrader, *Burkholderia multivorans* (NG1). Briefly, Triton X-100 enhanced bioavailability through emulsification and supported direct interfacial uptake, while the rhamnolipid mixture JBR-515 did not substantially emulsify hydrocarbons, enhancing bioavailability instead through micellar solubilization (Mohanty and Mukherji, 2013).

In *P. aeruginosa*, it has been observed that the uptake of rhamolipid-coated hexadecane droplets occurred through a mechanism very similar to pinocytosis (Cameotra and Singh, 2009); the latter can be tentatively defined here as “internalization of biosurfactant layered hydrocarbon droplet.” Depending on the physiology of the organism with respect to its preferred hydrocarbon accession mode (direct contact with sparingly soluble hydrocarbons, direct attachment to insoluble hydrocarbon droplets, micellization of hydrocarbons with biosurfactants), the presence of biologically derived and synthetic surfactants may inhibit biodegradation. Micelle cores can trap organic contaminants, creating a hydrophilic barrier between “hydrophobic microorganisms” and organic molecules, the result of which is the potential substrate becoming less available (Colores et al., 2000). Crucially, some microorganisms can emulsify hydrocarbons even in the absence of cell growth or uptake of hydrocarbons. That suggests that emulsification may be associated with the surface properties of the cells, because of attachment to the oil–water interface by general hydrophobic interactions rather than specific recognition of the substrate. Therefore, microbial cells may behave as fine solid particles at interfaces. Having knowledge of that, prompt the hypothesis that intact, stationary-phase microorganisms, referred previously as “hydrophobic” can stabilize oil–water emulsions by adhering to the oil–water interface a property related to cell surface hydrophobicity.

In mixed microbial communities, *in situ* production of microbial- or plant-derived biosurfactants, or exogenously added (bio)surfactants, may serve as a preferred substrate for a normally hydrocarbonoclastic species, limiting remediation outcomes (Franzetti et al., 2008b). Endogenous and exogenous biosurfactants may also prove toxic to some organisms by disrupting membrane permeability, interfering with chemotaxis-driven motility, and disrupting or limiting biofilm formation.

Biofilms, bacterial communities surrounded by self-produced polymeric matrices reversibly attached to an inert or a biotic surface (Costerton et al., 1995), are an adaptive mechanism for microorganisms to better cope with harsh physical and chemical conditions, to facilitate catabolite exchange, to increase horizontal gene transfer, and to regulate the redox state of their environment (Gorbushina and Broughton, 2009; Shemesh et al., 2010). Biofilm matrices may consist of extracellular polysaccharides (EPSs), proteins and DNA (Sutherland, 2001; Branda et al., 2005; Rinaudi and Gonzalez, 2009), with EPS affecting the porosity, density, water content, charge, hydrophobicity, and mechanical stability of biofilms (Flemming and Wingender, 2010). Biofilms may also enhance PHC bioremediation processes by increasing pollutant availability (Wick et al., 2002; Johnsen and Karlson, 2004). The secretion of polymers is often correlated with establishment of the biofilm growth mode; thus, in case that secretion of polymers by microorganisms is followed by formation of biofilms on the surface of insoluble hydrocarbons, renders those microorganisms especially well-suited for the treatment of recalcitrant compounds because of their high microbial biomass within biofilm compared to the cells grown in dispersed culture along with their ability to immobilize compounds by biosorption. Moreover, the biofilm lifestyle facilitates degradation processes by maintaining optimal conditions of pH, localized solute concentrations and redox potential in the vicinity of the cells (Singh et al., 2006).

In addition to the production of biosurfactants and biofilm formation, chemotaxis, the targeted movement of microorganisms in response to chemical gradients with the aim of finding ideal conditions for growth and survival (Eisenbach and Caplan, 1998; Wadhams and Armitage, 2004; Baker et al., 2006a,b; Paul et al., 2006; Rao et al., 2008; Hazelbauer and Lai, 2010; Krell et al., 2011), has been shown to be important for microbial exploitation of PHCs in soil and water (Marx and Aitken, 2000; Pandey and Jain, 2002; Parales and Haddock, 2004; Ford and Harvey, 2007; Strobel et al., 2011). For example, the capability of bacteria to sense and swim toward *n*-hexadecane (Nisenbaum et al., 2013), gas oil (D’Ippolito et al., 2011), as well as various monocyclic and PAHs and their nitro-, amino-, or chloro-substituted relatives has been demonstrated to stimulate degradation of the corresponding PHCs (Grimm and Harwood, 1997; Parales et al., 2000; Samanta and Jain, 2000; Pandey et al., 2002; Lanfranconi et al., 2003; Law and Aitken, 2003; Ortega-Calvo et al., 2003; Vardar et al., 2005; Cunliffe et al., 2006; Gordillo et al., 2007; Iwaki et al., 2007; Peng et al., 2008; Bisht et al., 2010; Tremaroli et al., 2010; Fernandez-Luqueno et al., 2011), presumably by allowing the microorganism to balance access to substrate and substrate toxicity (Olson et al., 2004; Jeong et al., 2010).

In fact, the chemotactic behavior of bacteria can be either toward (positive chemotaxis) or away (negative) from the chemical gradient. Thus, chemotaxis presumably acts like a balance mechanism that helps the bacteria to perform in an ideal way if it increases bioavailability of pollutants whilst, at the same time protects them in case of toxicity. For example, this balance may explain why the naphthalene degrading *Pseudomonas putida* PpG7 was repelled by vapor-phase naphthalene at steady state gaseous concentrations that were significantly lower than the aqueous concentrations that resulted in positive chemotaxis (Hanzel et al., 2010).

In some cases, the chemotaxis mechanisms for PHC degrading microorganisms are well-characterized, and it has been observed that, in some cases, PHC catabolic genes are co-located with chemotaxis genes on plasmids (Grimm and Harwood, 1999). It has been shown in bacteria of the genus *Pseudomonas* that the chemotactic response is mediated by the McpT chemoreceptor encoded by the pGRT1 megaplasmid. Two alleles of *mcp*T are borne on this plasmid and inactivation of either one results in a loss of the chemotactic phenotype, while cloning of *mcp*T into a plasmid complemented not only the *mcp*T mutants, but also made it possible to transfer chemotactic response to other *Pseudomonas* strains for high PAH concentrations, indicating that chemotaxis toward toxic PAHs is gene-dose dependent (Lacal et al., 2011). Overall, increased expression of motility and chemotaxis genes suggest that microbial communities are able to ramp up metabolic pathways that will allow for direct contact with hydrocarbon compounds (Smith et al., 2013).

## Remediation Strategies

Historically, both *ex situ* and *in situ* bioremediation approaches have been used for the restoration of PHC-polluted environments (Stroud et al., 2007). However, *in situ* approaches have become more prevalent as costs compared to *ex situ* are generally lower with fewer disruptions to the natural landscape (Romantschuk et al., 2000; Jorgensen, 2007). The different approaches used for assessment of the ecological sustainability of *in situ* bioremediation processes have been thoroughly reviewed (Pandey et al., 2009), with natural attenuation (Smets and Pritchard, 2003; Scow and Hicks, 2005) and biostimulation/bioaugmentation being discussed below.

## Natural Attenuation

A growing body of studies, including modeling and field experimentation provide evidence that natural attenuation is a promising remediation option for soils, estuarine sediments and groundwater contaminated by PHCs (Khan and Husain, 2003; Suarez and Rifai, 2004; Verginelli and Baciocchi, 2013). In the same context, several other reports have underlined the significant role of subsurface natural attenuation processes in bioremediation (Pasteris et al., 2002; Devaull, 2007; Lundegard et al., 2008; Abreu et al., 2009). Natural attenuation has been shown as an effective bioremediation option for a chronically diesel-oil-polluted site over a long period of time under unfavorably cold conditions (Margesin and Schinner, 2001).

The recovery of the Gulf of Mexico after the Deepwater Horizon blowout testifies to the fact that *in situ* bioremediation based on natural attenuation can be successful after large scale spills. Indeed, quick adaptation of the native microflora of the deep sea ecosystem to oil contamination resulted in dominance of bacteria of the order *Oceanospirillales* in the γ*-Proteobacteria*, a group which includes known psychrophilic hydrocarbon degraders and microorganisms from hydrocarbon-dominated environments (Hazen et al., 2010).

## Biostimulation, Bioaugmentation, and Endophytes

The principle behind biostimulation as a method to increase PHC degradation relies on the establishment of a propitious environment for hydrocarbonclastic bacterial communities through the addition of nutrients (e.g., nitrogen and phosphorus, horse manure, poultry litter, domestic sewage, rice straw biochar, crop residues), and other supplementary components such as biosurfactants and electron acceptors [e.g., O_2_, chelated Fe (III), nitrates, sulfate] (Gallego et al., 2001; Molina-Barahona et al., 2004; Coles et al., 2009; Lai et al., 2009; Qin et al., 2013; Zhao et al., 2015; Ladino-Orjuela et al., 2016). The adjuvant role of these factors is related either to the metabolic activity of the naturally occurring degrading bacteria or to the bioavailability of PHCs. Among these biostimulants, addition of nutrients has been demonstrated to improve the degradation potential of native microbial communities (Thomassin-Lacroix et al., 2002; Delille et al., 2004; Garcia-Blanco et al., 2007). Studies at both laboratory and field scales have revealed enhanced degradation of PHCs (diesel oil, pyrene, phenanthrene) based on the addition of biosolids, inorganic fertilizers (rich in N and P) and organic fertilizers (Braddock et al., 1995; Carmichael and Pfaender, 1997; Margesin et al., 2003; Xu and Obbard, 2003; Sarkar et al., 2005).

Moreover, it has been observed that the higher the initial PHC contamination, the more marked was the effect of fertilization on PHC removal (Margesin et al., 2007). Similar results have been observed in aquatic environments, however, caution is required given that high nutrient levels can be the causative agent of ecological impairments such as eutrophication (Nikolopoulou and Kalogerakis, 2009).

Approximately, 1–5% N by weight of oil with a ratio of N:P between 5 and 10:1 is applicable for oil spill remediation (Swannell et al., 1996). Furthermore, based on a theoretical calculation the conversion of 1 g of hydrocarbon to cell materials requires the utilization of 150 mg of nitrogen and 30 mg of phosphorus (Rosenberg and Ron, 1996).

A number of comparative studies have reported different C:N:P ratios as the most suitable prior to the commencement of *in situ* bioremediation. In this sense, it has been proposed that optimal C:N:P mole-ratios to enhance hydrocarbon removal in soil are at the levels of 100:9:2, 100:10:1, 100:10:5, or 250:10:3 (Zawierucha and Malina, 2011).

Given that most energetically favorable terminal electron acceptor is O_2_, it is assumed that adequate aeration through mechanical tillage, forced aeration and addition of alternative oxygen sources, such as oxygen-releasing compounds (ORCs), or agents such as potassium permanganate (KMnO_4_), hydrogen peroxide (H_2_O_2_), or ozone (O_3_) should stimulate microbial activity and enhance aerobic biodegradation rates (Brown et al., 2003; Saito and Magara, 2003; Goi et al., 2006; Menendez-Vega et al., 2007; Tsai and Kao, 2009).

Furthermore, the rate of hydrocarbon removal has also been stimulated by generating optimal conditions for other physical factors such as temperature (Horel and Schiewer, 2009) and moisture (Zawierucha and Malina, 2011). Recently, the application of non-conventional biostimulation methods has been reported. For example, incorporating modified Fenton’s reagent as a pre-treatment in combination with inorganic fertilizers has improved the bioremediation of diesel polluted soil (Andrea Silva-Castro et al., 2013).

Several authors have investigated the impacts of *in situ* biostimulation treatments on bacterial diversity aiming to understand the relationships between the dominance, physiology and function of specific genera able to degrade contaminants of concern (Iwamoto et al., 2000; Evans et al., 2004). These observations suggest that identifying the key players that drive community structure is a prerequisite to comprehend, model, forecast, monitor, and control biostimulation processes (Hazen, 2010).

Another variant of bioremediation, bioaugmentation, involves the introduction in adequate numbers of bacterial populations with the necessary catabolic potential to mediate PHC degradation (Vogel, 1996; Paliwal et al., 2012). Therefore, selection and addition of (a) a pre-adapted bacterial strain, (b) a pre-adapted consortium, (c) genetically engineered bacteria, or (d) catabolic genes packaged in a vector to be transferred by conjugation into indigenous microorganisms, is of paramount importance for any bioaugmentation process (El Fantroussi and Agathos, 2005; Singer et al., 2005; Thompson et al., 2005). When considering bioaugmentation, it is important to consult local regulations and decide if: (1) a single strain or a known mixed microbial consortium can be introduced, (2) an autochthonous, defined as an indigenous bacterial consortium previously enriched from the polluted soil and cultivated with hydrocarbons as the carbon source can be re-inoculated or (3) an allochthonous, defined as a foreign consortium previously drawn from another PHCs polluted site, can be used (Ueno et al., 2007). In fact, the bioremediation of soils freshly contaminated with petroleum constituents could benefit from the addition of biota primed for PHCs biodegradation (Greenwood et al., 2009). Interestingly, based on the use of selected native strains, bioaugmentation has been shown to accelerate the bioremediation of soils co-contaminated with diesel oil and various heavy metals (Alisi et al., 2009). A study conducted to evaluate the potential of indigenous and exogenous microorganisms for bioremediation of clayey and silty soils polluted with diesel oil revealed that a native consortium was the best option for remediating the silty soil, while a combination of native and exogenous consortia was more effective for remediating the clayey soil (Moliterni et al., 2012). Most recently, the introduction of an exogenous PHCs-degrading consortium consisting of *Rhodococcus equi, Enterobacter* sp., *Acinetobacter calcoaceticus, Comamonas* sp., and *Pseudomonas alcaligenes*, increased the production of high erucic acid rapeseed (*Brassica napus*) biomass in soils treated with diesel oil ranging from 6,000 to 24,000 mg kg^-1^ dry soil (Graj et al., 2013). Despite the satisfactory nature of these experiments, the Achilles’ heel of traditional bioaugmentation remains if foreign bacteria are able to establish stable communities in competitive environments. In more detail, the exogenous introduction (bioaugmentation) of efficient PHCs degraders is actually a rational re-arrangement of the microbial richness aiming to the dominance of bacterial group(s) with specific catabolic traits necessary for the clean-up.

Thus, the diverse natural life forms that live in communities within the biotope inoculated with an exogenous inoculum, represents a major obstacle in the successful remediation performance of such an inoculum. Overviewing the literature, there is a consensus that the decline in population size of active exogenously inoculated bacteria is attributed to various factors of which competition with autochthonous bacteria for nutrients and electron acceptors seems to be paramount. Therefore, the long term efficacy of such inoculum requisites a successful initial establishment (Goldstein et al., 1985; van Veen et al., 1997; Bouchez et al., 2000; El Fantroussi and Agathos, 2005; Thompson et al., 2005).

Numerous studies have concluded that bioaugmentation through isolation and reintroduction of hydrocarbon degrading bacteria from a contaminated site is more effective than *in situ* biostimulation and natural attenuation when applied to sites contaminated with various PHCs (Bento et al., 2005; Smith et al., 2005; Liu et al., 2008; Couto et al., 2010).

However, it is often found that biostimulation with a commercial fertilizer is more effective than bioaugmentation (Demque et al., 1997), or that fertilizer effects of foreign inoculants are more important than the inoculants themselves.

While it may be possible to adjust the makeup of a microbial community, as was done with bioaugmentation of a bench scale biobarrier (Daghio et al., 2015) and nutrient addition to a diesel-contaminated boreal forest soil (Kauppi et al., 2011), PHC removal efficiencies may not be increased, although outcomes are site specific (Yergeau et al., 2009).

Bioaugmentation with endophytic bacteria with biodegradative capabilities may have benefits compared to conventional bioaugmentation with free-living bacteria, as endophytes may have greater potential to find a suitable niche in an established community due to their association with a plant host. Further benefits can be achieved if the endophyte transfers metabolic genes for biodegradation to native endophytes (Taghavi et al., 2005).

For example, *in situ* bioaugmentation by *P. putida* W619 decreased trichloroethylene evapotranspiration up to 90% under field conditions (Weyens et al., 2009a). This result was achieved after the establishment and enrichment of *P. putida* W619-TCE as a poplar root endophyte followed by further horizontal gene transfer of TCE metabolic activity to members of the poplar’s endogenous endophytic community (Weyens et al., 2009b).

**FIGURE 2:**
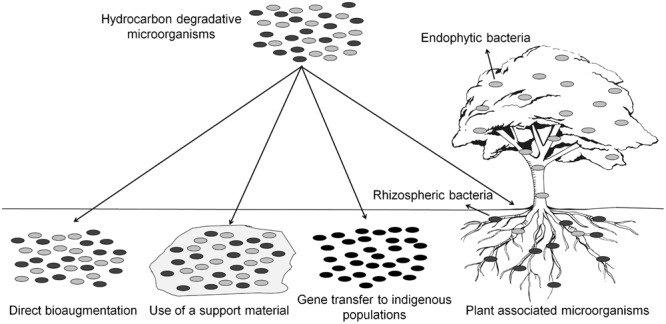
**Possible strategies for the bioremediation of PHC contaminated sites**.

For more information about the different techniques developed for bioaugmenting environmental sites (**Figure 2**), with emphasis on PHC spills, the reader is referred to the reviews of Gentry et al. (2004), Hosokawa et al. (2009), and Tyagi et al. (2011).

In addition, both bioaugmentation and biostimulation appear to be effective for enhancing PHC biodegradation in soil and, in some cases, the simultaneous application of these techniques results in additional improvement (Hamdi et al., 2007a; Mrozik and Piotrowska-Seget, 2010; Xu and Lu, 2010; Sun et al., 2012; Taccari et al., 2012). For example, it has been demonstrated that the highest pyrene removal (84%) was obtained through a combined bioaugmentation-biostimulation process, followed by bioaugmentation (57%), biostimulation (50%), and control (37%) processes (Ghaly et al., 2013).

Overall, site conditions, composition of the indigenous microbial community, and the type, quantity and toxicity of the pollutant present demand a case by case approach to deal with contamination challenges.

## Genes and Enzymes Participating in Aerobic Degradation of Hydrocarbons

In addition to promoting bioavailability (e.g., by addition or production of biosurfactants), and stimulating microbial activity (e.g., by biostimulation or bioaugmentation), PHC bioremediation can be further optimized by involving assiduously characterized bacterial strains carrying the necessary metabolic pathways for the complete degradation (mineralization) of components in petroleum mixtures.

**FIGURE 3:**
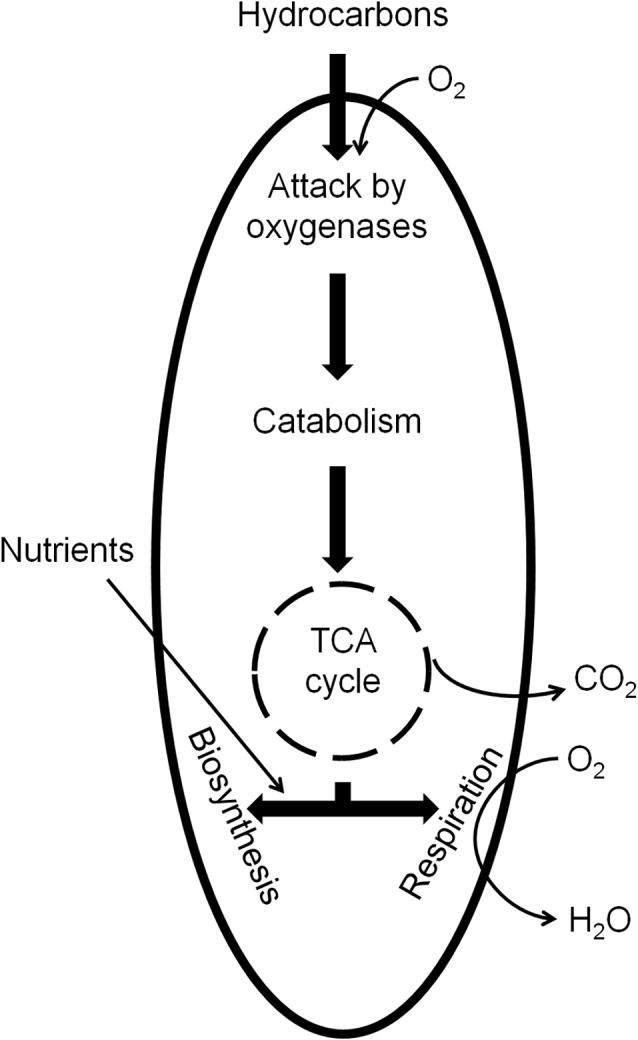
**Aerobic catabolism of PHCs by microorganisms**.

In general, even though the biodegradation of PHCs can occur under anaerobic conditions, the majority of them are more efficiently metabolized under aerobic conditions. **Figure 3** illustrates the basic principle of aerobic catabolism of PHCs. PHC biodegradability tends to decrease in the following order: *n*-alkanes > branched-chain alkanes > branched alkenes > low-molecular-weight *n-*alkyl aromatics > monoaromatics > cyclic alkanes > PAHs > asphaltenes (Atlas, 1981; Van Hamme et al., 2003; Tyagi et al., 2011). Despite the chemical stability of alkane molecules, in the presence of O_2_ they can be activated by oxygenases and completely oxidized to carbon dioxide and water.

The expression of genes involved in alkane degradation is strictly controlled (Wang and Shao, 2013), and microorganisms have multiple alkane degradation systems that target alkanes of different chain lengths (**Table 1**). Specific regulation mechanisms ensure that the genes involved in alkane degradation are expressed only under certain conditions, in the presence of the appropriate alkanes when other preferred substrates are not available (Rojo, 2009).

**Table 1:** Overview of the genes and enzymes involved in alkanes degradation listed in this review.

Gene/enzyme	Microorganisms	Reference
sMMO	*Methylosinus trichosporium* OB3b	Baik et al., 2003
sMMO	*Methylococcus capsulatus* (Bath)	Baik et al., 2003
pMMO	*Methylococcus capsulatus* (Bath)	Lieberman et al., 2003
*alkB1*	*Pseudomonas aeruginosa* PAO1	Marin et al., 2003
*alkB2*	*Pseudomonas aeruginosa* PAO1	Marin et al., 2003
*alkB1*	*Pseudomonas aeruginosa* RR1	Marin et al., 2003
*alkB2*	*Pseudomonas aeruginosa* RR1	Marin et al., 2003
*alkB1*	*Alcanivorax borkumensis* AP1	van Beilen et al., 2004
*alkB2*	*Alcanivorax borkumensis* AP1	van Beilen et al., 2004
P450-1	*Alcanivorax borkumensis* SK2	Schneiker et al., 2006
P450-2	*Alcanivorax borkumensis* SK2	Schneiker et al., 2006
P450-3	*Alcanivorax borkumensis* SK2	Schneiker et al., 2006
*alkB1*	*Alcanivorax hongdengensis* A-11-3	Wang and Shao, 2012
*alkB2*	*Alcanivorax hongdengensis* A-11-3	Wang and Shao, 2012
*p450-1*	*Alcanivorax hongdengensis* A-11-3	Wang and Shao, 2012
*p450-2*	*Alcanivorax hongdengensis* A-11-3	Wang and Shao, 2012
*p450-3*	*Alcanivorax hongdengensis* A-11-3	Wang and Shao, 2012
*AlkMa*	*Acinetobacter* sp. M-1	Tani et al., 2001
*AlkMb*	*Acinetobacter* sp. M-1	Tani et al., 2001
*almA*	*Alcanivorax dieselolei* B5	Wang and Shao, 2014
AlmA	*Acinetobacter* strain DSM 17874	Throne-Holst et al., 2007
LadA	*Geobacillus thermodenitrificans* NG80-2	Feng et al., 2007
sMMO	*Gordonia* sp. TY-5	Kotani et al., 2003
sBMO	*Pseudomonas butanovora*	Sluis et al., 2002
CYP153	*Dietzia* sp. Strain DQ12-45-1b	Liang et al., 2016
*alkB*	*Pseudomonas putida* GPo1	van Beilen et al., 2001
AlkB	*Gordonia* strain SoCg	Lo Piccolo et al., 2011
CYP153	*Acinetobacter* sp. EB104	Maier et al., 2001
P450	*Alcanivorax dieselolei* B-5	Liu et al., 2011
P450	*Rhodococcus erythropolis* strain PR4	Sekine et al., 2006

Generally, alkane-degradation by bacteria begins with an oxidative attack at the terminal methyl group with the formation of a fatty alcohol, aldehyde, and fatty acid. The carboxylic acid can then be combined with CoA and, via ß-oxidation, yield acetyl-CoA that enters the tricarboxylic acid (TCA) cycle. For short-chain length (C1–C4) *n-*alkanes, methane monooxygenases (MMO) are the first enzymes involved in the process. The MMO enzyme family consists of two distinct forms: a soluble di-iron methane monooxygenase (sMMO) and a membrane-bound copper-containing methane monooxygenase (pMMO); the alpha subunits of these enzymes are encoded by *mmoX* and *pmoA* genes, respectively. Notably, sMMO performs the co-oxidation of saturated, unsaturated, linear, branched and cyclic hydrocarbons, whereas pMMO has a much narrower substrate range, being mostly active against alkanes and alkenes with lengths up to five carbons (Berthe-Corti and Bruns, 2001 ; Steinkamp et al., 2001; Baik et al., 2003; Lieberman et al., 2003; Hua et al., 2011; Jiang et al., 2011). Gaseous alkanes are metabolized by strains expressing propane or butane monooxygenases (BMOs) that are related to pMMO or sMMO, respectively. For example, *Gordonia* sp. TY-5 has been reported to be able to use propane as the sole carbon source, but no other gaseous alkanes. A complete operon encoding for PmA, which is similar to the α subunit of sMMO, an NADH-dependent reductase and a regulatory protein, was cloned and sequenced from this strain. Upon deletion of one of the subunits, the ability of the organism to grow on propane was nullified, corroborating its role in propane oxidation (Kotani et al., 2003). The hydroxylase subunits of propane monooxygenase show relatively high sequence similarity with butane monooxygenase (sBMO) isolated from *Pseudomonas butanovora*, an organism which oxidizes butane to 1-butanol. This BMO has been cloned and is similar to sMMO: the hydroxylase subunits α and ß, and the regulatory protein B show more than 60%, 50% amino acid sequence identity, respectively, to the corresponding subunits of sMMOs (Sluis et al., 2002). The differential regulation of multiple alkane hydroxylases has been described in *P. aeruginosa* RR1 and in *P. aeruginosa* PAO1. These strains contain the alkane hydroxylases AlkB1 (which oxidizes C16–C24 *n-*alkanes) and AlkB2 (which oxidizes C12–C20 *n*-alkanes). When C10–C22 alkanes are present, both genes are expressed but the expression of *alkB1* is double that of *alkB2*. Furthermore, *alkB2* is preferentially induced at the beginning of the exponential phase, and *alkB1* is preferentially induced during the late exponential phase, with expression of both genes decreasing during the stationary phase (Marin et al., 2003).

A more complex system has been described in *Alcanivorax borkumensis*, an organism with two alkane hydroxylases (AlkB1, active on C5–C12 *n*-alkanes and AklB2, active on C8–C16 *n*-alkanes) and three cytochrome P450s involved in alkane oxidation (P450-1, P450-2, and P450-3) (van Beilen et al., 2004; Schneiker et al., 2006). The expression of *alkB1* and *alkB2* genes is induced when C10–C16 alkanes are provided and decreases when the cells enter the stationary phase (van Beilen et al., 2004; Sabirova et al., 2006; Schneiker et al., 2006). An AlkS-like activator seems to be involved in the activation of *alkB1* in response to the presence of alkanes. Higher levels of AlkS have been detected when hexadecane was provided instead of pyruvate, and the *alkB1* promoter in *A. borkumensis* has an AlkS-binding site immediately upstream (van Beilen et al., 2004; Sabirova et al., 2006). A regulator of the AraC family is located close to P450-1, however, its role in the regulation of the expression of P450-1 still has to be investigated (Schneiker et al., 2006). It was recently suggested that a potential AraC family regulator (CypR) is involved in CYP153 gene activation, a gene that encodes an alkane hydroxylase that belongs to the cytochrome P450 superfamily (Funhoff et al., 2006) in the Gram-positive bacterium *Dietzia* sp. strain DQ12-45-1b (Liang et al., 2016). As in *A. borkumensis, Alcanivorax hongdengensis* degrades alkanes by using *alkB1, alkB2, p450-1, p450-2*, and *p450-3*. In *A. hongdengensis* a gene that encodes for a protein homologous to TetR family regulators is located downstream of *alkB1*. Furthermore, the presence of a regulator of the GntR family has been observed upstream of *alkB2* but its role in the regulation of the degradation pathways is still not known (Wang and Shao, 2012). *Acinetobacter* sp. M-1 has two alkane hydroxylases, AlkMa and AlkMb. *AlkMa* is induced by AlkRa in the presence of >C22 *n-*alkanes, and the *alkMb* gene is induced by AlkRb when C16–C22 *n-*alkanes are provided (Tani et al., 2001).

Other important mechanisms regulating alkane metabolism are product repression and catabolite repression control (Rojo, 2009). For example, expression of BMO in *P. butanovora* is repressed by propionate, a downstream metabolite of propane oxidation (Doughty et al., 2006). Moreover, propionate acts as a repressor of alkane degradation in *P. butanovora* by competitive inhibition for the BMO catalytic site (Doughty et al., 2007). It has been shown that expression of BMO-encoding genes is activated by the putative sigma (54)-transcriptional regulator BmoR. This peptide recognizes alcohols and aldehydes produced during alkane degradation (Kurth et al., 2008).

**FIGURE 4:**
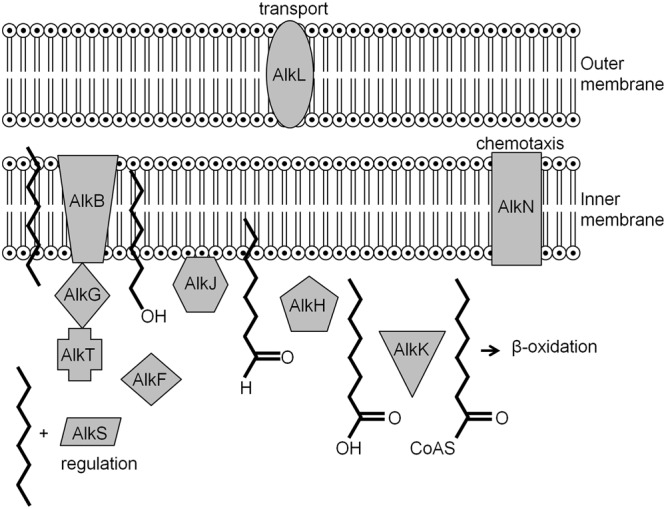
**Locations and functions of the *alk* gene products in the inner and outer membrane of gram negative bacteria (Van Hamme et al., 2003)**.

In microorganisms that are versatile with respect to PHC metabolism can be repressed in the presence of other carbon sources that are used as preferred substrates via catabolite repression (Rojo, 2010). As an example, the most thoroughly characterized alkane degradation pathway, encoded by the OCT plasmid carried by *P. putida* GPo1 (van Beilen et al., 2001), will be described. In this system, the *alkBFGHJKL* operon encodes the enzymes necessary for converting alkanes into acetyl-coenzyme A (CoA), while *alkST* encodes a rubredoxin reductase (AlkT) and the positive regulator for the *alkBFGHJKL* operon (AlkS). These two operons are located end to end, separated by 9.7 kb of DNA, within which lies *alkN*, a gene coding for a methyl accepting transducer protein that may be involved in alkane chemotaxis. When alkanes are provided, the transcriptional regulator AlkS activates *alkST* gene expression by using the *PalkS2* promoter (Canosa et al., 2000). Increased AlkS levels activate expression of *alkBFGHJKL* via the *PalkB* promoter (Canosa et al., 1999; Panke et al., 1999). However, when the cells are growing in a rich medium, the activation of both *PalkB* and *PalkS2* is negatively affected even if alkanes are provided (Yuste et al., 1998; Staijen et al., 1999; Canosa et al., 2000). In a rich medium the global regulatory protein Crc (catabolite repression control) inhibits translation of *alkS* mRNA (Moreno et al., 2007). It was suggested that Crc and the protein Hfq form a stable complex with RNA resulting in the inhibition of translation initiation (Moreno et al., 2015). It has been demonstrated that in *P. putida*, Crc also limits the translation of mRNAs coding for enzymes involved in the first steps of alkane degradation (Hernandez-Arranz et al., 2013). Another regulation system involves the cytochrome *o* ubiquinol oxidase (Cyo), a component of the electron transport chain (Dinamarca et al., 2002). The expression of *cyo* depends on the oxygen concentration and the presence of the carbon source, with Cyo levels being correlated with repression of alkane degradation (Dinamarca et al., 2002, 2003). The role of Cyo during the degradation of long chain alkanes in *Alcanivorax dieselolei* has been reported (Wang and Shao, 2014). In the presence of long chain alkanes and pristane, Cyo was expressed resulting in decreased AlmR production. AlmR is a negative regulatory protein of *almA*, a gene which encodes for the AlmA hydroxylase that is active against both long chain and branched alkanes (Wang and Shao, 2014). Noteworthy, at this point is that of all the genes mentioned, the function of *alkL* remains unknown, although, it is suspected to be involved in transport (**Figure 4**).

Another class of hydroxylases, facilitating the terminal hydroxylation of medium-chain *n-*alkanes, includes enzymes related to the soluble cytochrome P450 CYP153 from *Acinetobacter* sp. EB104 (Maier et al., 2001). Since that enzyme was characterized, several researchers have reported that bacteria belonging to *Mycobacterium, Rhodococcus*, and *Alcanivorax* isolated from various environments such as contaminated soil, groundwater and surface water, use that enzymatic machinery to degrade medium-chain alkanes (Kubota et al., 2005; Schneiker et al., 2006; Sekine et al., 2006; Wang et al., 2010; Liu et al., 2011). Even though assimilation of alkanes up to C20 is reported for bacteria containing AlkB family and cytochrome P450 alkane hydroxylases, there is a scarcity of information on metabolic pathways and enzyme systems that degrade >C20 alkanes (Rojo, 2009).

Usually, the alkane hydroxylases present in bacteria able to degrade alkanes longer than C20 are not evolutionary related to known AlkB and P450-like proteins and include AlmA (a flavin binding monooxygenase involved in the degradation of long-chain *n*-alkanes of C32 and longer) from *Acinetobacter* strain DSM 17874 (Throne-Holst et al., 2007), and LadA from *Geobacillus thermodenitrificans* NG80-2 (Feng et al., 2007), able to generate primary alcohols from C15 to C36 alkanes. *Acinetobacter* sp. M-1 (Sakai et al., 1994), and *Acinetobacter baylyi* ADP1 (Vaneechoutte et al., 2006), have been also found to grow with C32 and C36, respectively.

In addition, long chain *n*-alkane degrading bacterial species such as: *Marinobacter aquaeolei* VT8, *Oceanobacter* sp. RED65, *Ralstonia* spp., *Mycobacterium* spp., *Photorhabdus* sp., *Psychrobacter* spp., and *Nocardia farcinica* IFM10152, has been reported (Wentzel et al., 2007). Lately, a unique functional AlkB-type alkane hydroxylase system has been described that allows growth on long-chain liquid and solid *n*-alkanes in the Gram-positive *Gordonia* strain SoCg (Lo Piccolo et al., 2011). In contrast to alkanes, the general mode of monoaromatic and PAH biodegradation requires the presence of bacteria that harbor catabolic genes coding for dioxygenases. Generally, catabolism of PAHs is triggered by a dioxygenase reaction that adds hydroxyl groups (OH) to one ring.

Thereafter, the hydroxylated ring is subjected to ring fission, producing a substituted PAH with one ring less than the parent molecule. Subsequent oxygenase reactions are utilized to ultimately mineralize the PAH (Olson et al., 2003). Ring-hydroxylating dioxygenases related to polycyclic aromatic hydrocarbon oxidation (PAH-RHD), such as those encoded by the *nah, nod*, and *phn* genes in Gram-negative bacteria, and the evolutionarily correlated *nid, nir*, and *nar* genes in Gram-positive bacteria, catalyze the first step of the PAH degradation pathway (Larkin et al., 1999; Saito et al., 2000; Khan et al., 2001). In this step, dioxygenase-catalyzed oxidation of arenes yields vicinal *cis*-dihydrodiols as the early bioproducts of a multicomponent enzyme system.

Furthermore, these di-hydroxylated intermediates may then be cleaved by intradiol or extradiol ring-cleaving dioxygenases through either an ortho-cleavage pathway or a meta-cleavage pathway, leading to central intermediates such as protocatechuates and catechols that are further converted to TCA cycle intermediates (Peng et al., 2008). The catalytic component with hydroxylase activity is composed of an alpha subunit of about 50 kDa and a beta subunit of 20 kDa, which assemble in a α3ß3 heterohexamer.

Each alpha subunit consists of two domains, the N-terminal Rieske domain, which contains a [2Fe-2S] cluster, and the C-terminal catalytic domain, which contains a mononuclear ferrous ion close to the substrate-binding site. The catalytic component requires electrons to activate oxygen at each cycle of hydroxylation of the substrate. Two auxiliary proteins, a ferredoxin and a flavin-containing oxidoreductase, often provide the necessary reductant at the expense of NAD(P)H oxidation (Jouanneau et al., 2011). Genes coding for the catalytic domain of PAH-RHDs (α-subunit) have been broadly used as biomarkers of PAH-degrading potential in various environments, making this subunit a valuable tool for studying RHD biodiversity (Flocco et al., 2009; Ding et al., 2010).

Based on amino acid sequence comparisons of the catalytic oxygenase α subunits, four discernible classes have been reported. These are: (a) the naphthalene family which includes Gram-negative bacterial enzymes responsible for the degradation of naphthalene and phenanthrene; (b) the benzoate family encompassing enzymes for the oxidation of aromatic acids; (c) the phthalate class that includes the diverse mono- and dioxygenases (interestingly the majority of the members of this family lack the ß subunits and possess only the reductase component in the electron transport chain); and (d) the toluene/biphenyl class that contains enzymes from both Gram-negative and Gram-positive microbes capable of transforming toluene, benzene, and chlorobenzenes (Gibson and Parales, 2000).

Historically, the critical point for the analysis of PAH degradation by aerobic bacteria started with the discovery, in *P. putida* strain G7, of naphthalene catabolic genes (*nah*) located on the plasmid NAH7 (Simon et al., 1993). After that discovery, work mainly on *Pseudomonas* species made evident that naphthalene biodegradation occurs via the formation of salicylate as an intermediate.

Upon examination of the diversity of dioxygenases involved in the degradation of low molecular weight (LMW) and high molecular weight (HMW) PAH compounds (e.g., naphthalene, phenanthrene, anthracene, pyrene, benzo[a]pyrene, benzo[a]anthracene), it is noticeable that both Gram-negative genera like *Pseudomonas, Ochrobactrum, Polaromonas, Sphingomonas, Novosphingobium, Acidovorax* and *Burkholderia*, and Gram-positive genera like *Mycobacterium, Gordonia, Bacillus, Nocardia*, and *Rhodococcus*, are exploiting these enzymes for the degradation of the aforementioned compounds (**Table 2**). Overall, the oxidation of naphthalene follows either the gentisic acid (Grund et al., 1992), or catechol (ortho and/or meta) degradation pathways (Eaton and Chapman, 1992) in order to generate compounds for integration in the TCA cycle, and there is a good body of evidence linking stimulated microbial PHC biodegradation to the presence of plant metabolites in the rhizophere as discussed in the next sections.

**Table 2:** Overview of the genes and enzymes involved in PAH degradation listed in this review.

Gene/enzyme	Microorganisms	Reference
*nah*	*Mycobacterium* sp. strain PYR-1	Khan et al., 2001
*nod*	*Rhodococcus* sp. strain NCIMB12038	Larkin et al., 1999
*phn*	*Nocardioides* sp. strain KP7	Saito et al., 2000
*nidA*	*Rhodococcus wratislaviensis* IFP 2016	Auffret et al., 2009
*nah*	*Pseudomonas stutzeri* AN10	Bosch et al., 2000
*nid*	*Mycobacterium* spp.	Brezna et al., 2003
2-Carboxybenzaldehyde dehydrogenase	*Nocardioides* sp. strain KP7	Iwabuchi and Harayama, 1997
α-Subunit of the polycyclic aromatic hydrocarbon ring-hydroxylating dioxygenases (PAH-RHDα)	*Pseudomonas, Polaromonas, Sphingomonas, Acidovorax, Burkholderia, Mycobacterium, Gordonia, Terrabacter, Nocardioides*, and *Bacillus*	Jurelevicius et al., 2012
*nar*B	*Rhodococcus* sp. NCIMB12038	Kulakov et al., 2000
Gentisate 1,2-dioxygenase	*Polaromonas naphthalenivorans* CJ2	Lee et al., 2011
Catechol 2,3-dioxygenase	*Burkholderia* sp. AA1	Ma and Herson, 2000
β-Ketoadipate and gentisate pathways	*Polaromonas* sp. strain JS666	Mattes et al., 2008
*phn*	*Sphingomonas* sp. strain LH128	Schuler et al., 2009
Catechol 1,2-dioxygenase and catechol 2,3-dioxygenase	*Gordonia polyisoprenivorans*	Silva et al., 2012
Catechol dioxygenases	*Pseudomonas* sp., *Ochrobactrum* sp., *Rhodococcus* sp.	Singh et al., 2013
*phn* genes	*Acidovorax* sp.	Singleton et al., 2009
*nidA, bphA3A4C*	*Novosphingobium* sp. PCY, *Microbacterium* sp. BPW, *Ralstonia* sp. BPH, *Alcaligenes* sp. SSK1B, *Achromobacter* sp. SSK4	Wongwongsee et al., 2013
1,2-Dihydroxynaphthalene oxygenase	*Rhodococcus* sp. strain b4	Grund et al., 1992
*nah*	*Pseudomonas aeruginosa* PAO1	Eaton and Chapman, 1992

## Plants and Bacteria for the Remediation of Petroleum Hydrocarbons

Phytoremediation, defined as the use of plants and their associated microorganisms to assimilate, transform, metabolize, detoxify and degrade various toxic inorganic and organic compounds (e.g., PHCs, pesticides, dyes, solvents) found in soil, water, groundwater, and air is generally considered as an environmentally friendly, cost effective, and socially accepted remediation approach (Salt et al., 1995, 1998; Alkorta and Garbisu, 2001; Pilon-Smits, 2005; Sandhu et al., 2007; Reichenauer and Germida, 2008;, Wenzel, 2009; Prasad et al., 2010; Kabra et al., 2012). For more information about the advantages and disadvantages of phytoremediation we refer to the following reviews (Susarla et al., 2002; Kuiper et al., 2004; Arthur et al., 2005; Pandey et al., 2009 ; Vangronsveld et al., 2009).

Plant-associated bacteria include endophytic, phyllospheric and rhizospheric bacteria, and they have a variety of interactions with plants, ranging from being active pathogens, opportunistic pathogens, and bacteria that dwell within the plant and merit some physical protection, to bacteria actively interacting with the host plant generating mutually beneficial association for both organisms (Newman and Reynolds, 2004; Weyens et al., 2009c). The ability of bacteria to degrade PHCs is attributed to the presence of catabolic genes and enzymes, which allow them to utilize the complex chemicals found in petroleum mixtures as vital energy sources (Rojo, 2009; Das and Chandran, 2010). Many bacterial strains have been reported to encompass the metabolic pathways required for the degradation of the relevant hydrocarbons. Species of *Pseudomonas, Acinetobacter, Mycobacterium, Haemophilus, Rhodococcus, Paenibacillus*, and *Ralstonia* belong to the most extensively studied bacteria (Tyagi et al., 2011). On the other hand, though a substantial number of hydrocarbons can be metabolized by bacteria, in the absence of plants this process is not always efficient due to the relatively low number of these microorganisms in bulk soil. Indeed, in the rhizosphere 10–1000 times higher microbial activity has been reported. Hence, the role of plants in the ongoing process is equally important (Palmroth et al., 2002; Gaskin et al., 2008).

In another context, PHCs are giving rise as serious threat not only to soil but also to estuarine sediments (Chapman and Wang, 2001; Daane et al., 2001). The ecological importance of these ecosystems, along with their susceptibility to pollutants such as PHCs (Andrade et al., 2004), have fostered various research groups to investigate, whether plant–microorganisms associations may actively contribute to PHC degradation in estuarine environments. In fact, a number of recent studies have evaluated the influence of different salt marsh plant–bacteria associations on PHC fate and concluded that such symbiosis enhances significantly the degradation pattern via alteration of the functional diversity of the PHC degrading bacterial community (Oliveira et al., 2014, 2015).

Phytoremediation encompasses four distinct mechanisms namely phytostabilization, phytodegradation, phytovol-atilization, and rhizodegradation (Germida et al., 2002). Briefly, the term phytostabilization includes immobilization of the contaminants in soil, either simply by preventing erosion, leaching, or dispersion, or by transforming them through precipitation in the rhizosphere to less bioavailable forms. In an integrated approach phyto- and rhizodegradation can be approached as a mutually beneficial form of phytoremediation, where both plants and microorganisms mediate the breakdown of the contaminants via the use of their enzymatic machinery. Next phytovolatilization, due to the complete removal of the pollutant from the site as a gas, without further need for plant harvesting and disposal, holds promise as an attractive technology (Pilon-Smits, 2005; Lim et al., 2016).

In addition to the these concepts, a number of studies have shown that phyllosphere bacteria possess the ability to utilize gaseous and deposited PHCs (Waight et al., 2007; Yutthammo et al., 2010; Al-Awadhi et al., 2012; Ali et al., 2012); the latter holds great potential in air clean-up by opening up the new direction of air phyllo-remediation, which is actually the exploitation of air remediation capabilities based on the cooperation between plants and their associated phyllo-sphere microorganisms (Weyens et al., 2015).

Despite the fact of continuous exchange with airborne populations (Whipps et al., 2008), after recruitment phyllospheric bacteria are able to form real communities, prompting the hypothesis that they endure specific selection processes (Rastogi et al., 2012; Vorholt, 2012). The driving forces thought to govern community structure include plant species, leaf age, season, geographical location, and various environmental factors (Vokou et al., 2012; Muller and Ruppel, 2014). Thus, because of the high variability of phyllospheric community structure, further research about the bacterial communities hosted by different plant species in different environments is needed in order to evaluate their potential contribution to air bioremediation. Generally, in these very close plant–bacteria interactions, plants provide nutrients and residency for bacteria, which in exchange can improve applicability and efficiency of phytoremediation in case of sites contaminated by PHCs.

In a recent review (Thijs et al., 2016), it has been suggested that considering meta-organisms in their natural contexts (that is, the host and its microbiome together), will increase our knowledge of plant–microbial interactions and therefore facilitate translation to more effective, and predictable phytoremediation approaches. In the following sections, selected paradigms will be described to shed light to the field of PHC degradation via plants, bacteria, and their intimate interactions.

## Plants and PHC Remediation

In order to survive and thrive in PHC contaminated environments, plants must exhibit: (i) a tolerance to one or more components of petroleum mixtures, (ii) high competitiveness, (iii) fast growth, and (iv) the ability to produce and secrete hydrocarbon degrading enzymes. In this context, plants may be positively influenced by the presence of bacteria that are able to: synthesize plant hormones, such as, indole-3-acetic acid (IAA), gibberellins (GAs), and cytokinins (CKs); suppress ethylene production via 1-aminocyclopropane-1-carboxylate (ACC) deaminase activity; fix nitrogen; mobilize nutrients such as phosphorus and other minerals important in plant growth and development (Hardoim et al., 2008; Glick and Stearns, 2011); and metabolize a broach range of PHCs (Reed and Glick, 2005).

*In situ* implementation of phytoremediation strategies to restore contaminated sites has several drawbacks compared to traditional technologies such as pump and treat of contaminated groundwater, soil excavation and above-ground treatment. For example, if a plant has a shallow root zone and slow growth rates long periods of time may pass before contact with the target pollutant is made, if it is reached at all. The toxicity of the pollutants to native or introduced vegetation may result in inhibition of seed germination, reduced photosynthetic pigment production, compacted growth of tissues (root, aerial parts), slackening of nutrient assimilation and disruption of root architecture (Smith et al., 2006; Meudec et al., 2007; Euliss et al., 2008). Hence, selection of plants with increased pollutant tolerance, production of sufficient root and shoot biomass, suitability for various soil types, effective pollutant uptake mechanisms, and appropriate metabolic capabilities to degrade organic pollutants are prerequisites for successful remediation (Wenzel, 2009).

The initial physiological response of plants to PHCs in soil includes PHC uptake, translocation, and accumulation in organs such as roots and shoots. The rates of these processes are generally related to PHC concentration (Wild et al., 2005; Lu et al., 2010), lipophilicity, solubility, and volatility. Compound lipophilicity, expressed as an octanol-water partition coefficient (K_ow_), gives some indication about the tendency of a molecule to move through lipid bilayers, with log K_ow_ values between 0.5 and 3 reflecting compounds with sufficient hydrophobicity to move through membrane lipid bilayers while exhibiting sufficient water solubility to dissolve in cellular fluids (Cherian and Oliveira, 2005). Compounds with a log K_ow_ < 0,5 are characterized by high water-solubility, and plant roots do generally not translocate them at a rate surpassing passive influx (Cunningham and Berti, 1993), whereas compounds with a log K_ow_> 3.5 cannot be taken up and translocated into the plant due to tight sorption onto the soil and root surfaces (Meng et al., 2011).

After being transported inside the plant, PHCs can be either sequestered in root tissue, or transported into shoots and leaves, where they can be stored in vacuoles or volatilized into the atmosphere (Reichenauer and Germida, 2008).

Increasingly compelling evidence has accumulated about the use of plants for the remediation of environments polluted by PHCs (Liste and Alexander, 2000; van der Lelie et al., 2001; Newman and Reynolds, 2004; Pena-Castro et al., 2006; Euliss et al., 2008; Gerhardt et al., 2009; Peng et al., 2009; Zhang et al., 2012). Numerous studies focusing on plant species suitable for phytoremediation of PHC-contaminated soils have recognized that among others, Italian ryegrass (*Lolium perenne*), sorghum (*Sorghum bicolor*), maize (*Zea mays*), tall fescue (*Festuca arundinacea*), alfalfa (*Medicago sativa* var. Harpe), elephant grass (*Pennisetum purpureum*), bermuda grass (*Cynodon dactylon*), birdsfoot trefoil (*Lotus corniculatus* var. Leo), sunflower (*Helianthus annuus*), southern crabgrass (*Digitaria sanguinalis*), red clover (*Trifolium pratense*), beggar ticks (*Bidens cernua*), and sedge species (*Cyperus rotundus*) may be effective (Radwan et al., 1995; Wiltse et al., 1998; Chaineau et al., 2000; Huang et al., 2004; Parrish et al., 2004; Rutherford et al., 2005; Kaimi et al., 2007; Muratova et al., 2008; Shirdam et al., 2008; Ayotamuno et al., 2010; Tang et al., 2010; Yousaf et al., 2010; Hall et al., 2011; Basumatary et al., 2012, 2013). In general, the positive influence of leguminous plants is attributed in part to their ability to increase soil nitrogen concentrations in soils with high C:N ratio, whereas the positive contributions provided by grasses are correlated with their fibrous root systems, large root surface and deeper penetration into the soil matrix (Gaskin et al., 2008; Rezek et al., 2008). Taking into account the interplay between plants and their associate microorganisms in phytoremediaton, various research groups have investigated the role of fertilizers in this process and concluded that both the choice of, as well as the level of, added fertilizer is linked with the plant species present on site and the level of contamination (Cartmill et al., 2014; Jagtap et al., 2014; Ribeiro et al., 2014). It has been reported that the application of an ornamental plant (*Mirabilis jalapa*), characterized by non-trivial tolerance to petroleum contamination, strongly promoted PHC degradation when the concentration of PHC in soil was equal to or lower than 10,000 mg kg^-1^ (Peng et al., 2009).

Planting trees such as willows (*Salix* spp.) and hybrid poplars (*Populus* spp.) have been effective for remediating sites with contaminated groundwater (Cook et al., 2010) because they are easy to propagate, exhibit fast and perennial growth, generate phreatophytic roots that extend to the groundwater table, exhibit high water uptake rates, possess highly absorptive surface tissues, and are able to tolerate both a variety of contaminants and site flooding (Jordahl et al., 1997; Newman and Reynolds, 2004; Widdowson et al., 2005; Euliss et al., 2008; Barac et al., 2009).

The effects of varying concentrations of PHCs and nutrients on the spatial and temporal patterns of fine root production of hybrid poplar (*P. deltoides × P. petrowskyana* C. V. Griffin) has been investigated (Gunderson et al., 2008). It was observed that fine root production increased linearly up to approximately 500 mg kg^-1^ PHC, and then remained constant, and the working hypothesis is that the extensive fine root network may lead to enhanced contaminant degradation because of stimulated microbial activity due to a strong rhizosphere effect. A recent review compared the effectiveness of trees and grasses for remediation of PHCs and concluded that only minor differences are observed between trees and grasses with respect to average reduction of PHC concentrations (Cook and Hesterberg, 2013). Phytoremediation is a site-specific remediation method, explaining why contradictory results regarding the efficiency of this technology in removing contaminants from soil have been reported (Joner et al., 2004). Gaining knowledge about the molecular effects of PHCs on a range of plant species might contribute to better management of contaminated sites by providing physiological information to guide plant selection. In a recent study, aimed at unraveling PHC effects on plants, the global gene expression of 10-day-old *A. thaliana* seedlings exposed to the water-soluble fraction of a PHC mixture (WSF-MF380) was evaluated over time using whole genome microarray analysis. Results showed that the formation of an obstructive film covering the plant surface triggered gene expression responses similar to abiotic stresses such as heat, hypoxia, oxidative and osmotic stresses (Nardeli et al., 2016). Experiments with seedlings of *Amorpha fruticosa* exposed to PHC contaminated soil (≤15 g kg^-1^), demonstrated that the enzymes glutathione reductase (GR), superoxide dismutase (SOD) and catalase (CAT), effectively hampered reactive oxygen species (ROS) accumulation (Cui et al., 2016). The latter finding suggest the possibility of using the behavior of the antioxidant defense system and the growth reaction of seedlings under exposure to various PHCs concentrations as a valuable criterion for selection of the appropriate species for phytoremediation sites.

## Rhizosphere Bacteria and PHC Remediation

The photoautotrophic nature of plants, together with the fact that petroleum mixtures are poorly soluble in water means that for efficient PHC degradation the biocatalytic activities of rhizospheric microorganisms are essential. Generally, vegetated soils favor higher microbial numbers and diversity compared to bulk soil (Smalla et al., 2001; Haichar et al., 2008; Glick, 2010; Uroz et al., 2010). This effect is due to the release of organic compounds by plants commonly referred to as “rhizodeposits”; these compounds can be categorized as exudates, secretions, plant mucilages, mucigel, and root lysates (Olson et al., 2003) that are utilized by microorganisms as sources of carbon and energy (Chaudhry et al., 2005). Research has shown that plants, by releasing these organic compounds, change the physicochemical and biological properties of the soil most likely facilitating the attraction of chemotactic bacteria with desired metabolic activities (Hartmann et al., 2009). Plants release others organic compounds including terpenes, flavonoids and some lignin-derived components with chemical structures similar to those of PHCs, chemicals which may induce expression of PHC-degrading genes in rhizospheric microorganisms (Sun et al., 2010). Once attracted, PHC-degrading rhizosphere bacteria may ameliorate plant tolerance to PHCs and result in faster soil health recovery (Escalante-Espinosa et al., 2005; Barrutia et al., 2011). As an example, an increase of phenolic compounds found in root exudates has been associated with a higher degree of degradation of benzo[a]pyrene in the rhizosphere of *Phragmites australis* (Toyama et al., 2011).

More recently it has been demonstrated that PHC mineralization patterns by rhizosphere bacteria was substantially affected by root exudate composition. Specifically, certain compounds (e.g., acetate, alanine) were found to be associated with increased mineralization capacity, whilst others (e.g., malonate, trehalose, sucrose, glucose, xylose, mannose) resulted in decreased mineralization (Phillips et al., 2012).

A negative correlation in the degradation of PHCs (phenanthrene) and the presence of rhizodeposits (e.g., fumarate, mannitol, trehalose, sucrose, glucose, xylose, mannose, and fructose) in the rhizosphere of *Lolium multiflorum* has been demonstrated (Thomas and Cébron, 2016). Despite the divergent nature of these results, a vast body of literature confirms the beneficial association of bacteria and their host plants in the remediation PHCs at the level of the rhizosphere (**Table 3**). Root exudates may enhance microbial PHC metabolism in a number of ways: (i) PHC co-metabolism via plant secreted enzymes; (ii) increasing PHC bioavailability through the production of LMW carboxylates that may enhance PHC desorption and compete for soil adsorption sites (An et al., 2010; Gao et al., 2010), or through production of lipophilic or biosurfactant-like root exudates which may increase PHC solubility (Read et al., 2003); (iii) stimulation of microbial biomass and activity through excretion of labile C and N sources and by increasing nutrient availability due to the action of plant released enzymes (e.g., acid phosphatases) and organic chelators (Rohrbacher and St-Arnaud, 2016).

**Table 3:** Selected paradigms of successful rhizodegradation of PHCs listed in this review.

Plant species	Microorganisms	PHC-component	Reference
*Zea mays*	*Pseudomonas* sp. strain UG14Lr, *Pseudomonas putida* strain MUB1	Phenanthrene/pyrene	Chouychai et al., 2009, 2012
*Lolium perenne*	*Pantoea* sp. strain BTRH79	Diesel oil	Afzal et al., 2012
*Lotus corniculatus*	*Pantoea* sp. strain BTRH79	Diesel oil	Yousaf et al., 2010
*Medicago sativa*	*Rhizobium meliloti* strain ACCC 17519	Various PAHs	Teng et al., 2011
*Zea mays*	*Gordonia* sp. strain S2RP-17	Diesel oil	Hong et al., 2011
*Lolium multiflorum*	*Acinetobacter* sp.	Various PAHs	Yu et al., 2011
*Secale cereale, Medicago sativa*	*Azospirillum brasilense* strain SR80	Crude oil	Muratova et al., 2010
*Lolium multiflorum*	*Rhodococcus* sp. strain ITRH43	Diesel oil	Andria et al., 2009
*Sorghum bicolor*	*Sinorhizobium meliloti* strain P221	Phenanthrene	Muratova et al., 2009
*Hordeum vulgare*	*Mycobacterium* sp. strain KMS	Pyrene	Child et al., 2007a,b
*Triticum aestivum*	*Pseudomonas* sp. strain GF3	Phenanthrene	Sheng and Gong, 2006
*Trifolium repens*	*Rhizobium leguminosarum*	Chrysene	Johnson et al., 2004
*Hordeum vulgare*	*Pseudomonas fluorescens, Pseudomonas aureofaciens*	Phenanthrene	Anokhina et al., 2004
*Lolium multiflorum*	*Pseudmonas putida* strain PCL1444	Various PAHs	Kuiper et al., 2001
*Hordeum vulgare*	*Pseudomonas putida* strain KT2440	Various PAHs	Child et al., 2007a,b

## Endophytic Bacteria and PHC Remediation

Bacteria dwelling the internal tissues of plants (roots, stems, leaves) overcome some competition for nutrients and space experienced by rhizosphere bacteria, and are physically protected from unfavorable environmental conditions (Schulz et al., 2006).

Cultivable endophytic bacteria have been isolated from various plants species ranging from herbaceous crop plants such as sugar cane (Loiret et al., 2004), wheat (Larran et al., 2002), maize (Gutierrez-Zamora and Martınez-Romero, 2001), the metal hyperaccumulating alpine pennycress (*Thlaspi caerulescens*) (Lodewyckx et al., 2002), tall fescue (Malinowski et al., 2000), *Arabidopsis* seeds (Truyens et al., 2015a,b), different grass species (Dalton et al., 2004; Thijs et al., 2014b), woody tree species such as oak and ash (Weyens et al., 2009a), sycamore (Thijs et al., 2014a), poplar (Porteous Moore et al., 2006; Van der Lelie et al., 2009), *Mimosa pudica* (Pandey et al., 2005), pine seeds (Cankar et al., 2005), and other forest trees (Pirttilä and Frank, 2011).

Endophytic root colonization follows a general model where initially bacteria move toward the plant roots either passively via soil water fluxes, or actively via specific induction of flagellar activity by plant-released compounds. Subsequently, non-specific adsorption of bacteria to roots occurs, followed by anchoring that result in firm attachment to the root surface. Specific or complex interactions between the bacterium and the host plant, such as the secretion of root exudates, may arise resulting in changes in bacterial gene expression. Microscopic studies using gfp-labeled bacterial strains have illustrated this model in poplar trees (Germaine et al., 2004; Taghavi et al., 2009), and it has been observed that the phyllosphere may be a source of endophytic bacteria (Quadt-Hallmann et al., 1997).

In a pioneering study, it was shown that the enrichment of bacteria with the appropriate catabolic genes in the endophytic root compartment is correlated with the type and amount of contaminant and the genotype of the plant (Siciliano et al., 2001). Since then, a number of reports have confirmed that endophytic bacteria, have a better capacity to enhance PHC phytoremediation than rhizosphere or soil bacteria (Barac et al., 2004; Doty, 2008; Ryan et al., 2008; Weyens et al., 2010; Yousaf et al., 2011). This may be due to the fact that some endophytic bacteria have the potential to mineralize PHCs in trees, herbaceous plants and grasses (Barac et al., 2004; Phillips et al., 2008; Afzal et al., 2011). In a field experiment with four plant species, *Achillea millefolium, Solidago canadensis, Trifolium aureum*, and *Dactylis glomerata*, the presence of bacterial endophytes with PHC degradation capacity was observed (Lumactud et al., 2016). With the microbial communities, the class Actinobacteria was identified as the dominant group in three of the plant species examined, with Gammaproteobacteria being more abundant in *S. canadensis*.

Despite of the selective pressure of PHCs, the plant species remains the key factor shaping endophytic bacterial community structures. Ascertaining the specific interaction between plants and observed microbial phylotypes could generate critical information for the selection of optimized microbiomes with desirable host performance traits such as survival, growth, and fitness (Mueller and Sachs, 2015; Yergeau et al., 2015). Analysis of the microbiomes of two willow cultivars (*Salix purpurea* cv. Fish Creek, and *Salix miyabeana* cv. SX67) growing at different PHC concentrations demonstrated that increased concentrations of PHCs favored the abundance of root endophytes belonging to the Proteobacteria, particularly the classes Gammaproteobacteria and Alphaproteobacteria, while the Betaproteobacteria were predominant in the stems (Tardif et al., 2016). The Protoebacteria are a diverse group of organisms that include hydrocarbonoclasts and plant-growth promoting bacterial (PGPB) species (Bruto et al., 2014). It is not unlikely that some intrinsice host plant genotype-microbe signaling can favor the prevalence of these groups (Bulgarelli et al., 2012; Sessitsch et al., 2012).

Another contribution of endophytic bacteria to the overall PHC dissipation refers to their plant growth promoting traits, which facilitate the host’s performance by alleviating the stress encountered upon exposure to PHCs (Afzal et al., 2014). Genome sequence analysis of 56 endophytic/symbiotic Proteobacteria has provided useful insights about the molecular mechanisms that plant growth promoting endophytes exert on their hosts (Bruto et al., 2014). For example among the various direct and indirect mechanisms used by endophytic bacteria to aid their hosts in overcoming the toxic nature of PHCs, ACC – deaminase activity holds a pivotal role (Arshad et al., 2007; Afzal et al., 2013; Khan et al., 2013; Fatima et al., 2015).

With respect to the application of plant growth-promoting and PHC - degrading endophytes, a number of recent studies has identified bacterial isolates that may be useful inoculants to stimulate phytoremediation of PHC contaminated sites (Kukla et al., 2014; Tara et al., 2014; Zhang et al., 2014; Pawlik and Piotrowska-Seget, 2015; Balseiro-Romero et al., 2016).

## Conclusion and Future Perspectives

The use of PHCs has allowed for the development of privileged modern societies, with the associated cost of contaminated soil, seawater, freshwater and groundwater ecosystems. Given this, it is important to continue developing bio- and phyto-remediation approaches to deal with PHCs that are recalcitrant to metabolism because of their physico-chemical characteristics. Understanding plant-associated bacteria (endophytic, phyllospheric, and rhizospheric) and their varied interactions with plants (ranging from parasitism to mutualism) allows for an appreciation of the associations that have evolved between plants and bacteria to overcome constraints commonly found at contaminated sites.

The ability of bacteria to degrade PHCs is attributed to the presence of catabolic genes and enzymes, which allow them to utilize the complex chemicals found in petroleum mixtures for carbon and energy, an ability that can be enhanced by the presence of plants. Similarly, plants can be positively affected, directly or indirectly, by the presence of bacteria able to elicit drastic modifications in the health status of the plant via the synthesis of plant hormones, suppression of ethylene production, and the mobilization of otherwise unavailable nutrients.

While laboratory and field studies have indicated that bio- and phyto-remediation can be good treatment strategies for PHC polluted environments, more information is required to build accurate models for predicting treatment outcomes. Metagenomic, metatranscriptomic, metaproteomic, and metabolomic analyses of complex communities are allowing for a deeper understanding of how microbial communities interact with each other, the environment and the organisms around them (Villas-Boas and Bruheim, 2007; Bell et al., 2014; Kaul et al., 2016). It is easy to envision implementing metagenomic tools in the field of PHC remediation in order to: pre-assess the biodegradative capacity of an environment, monitor *in situ* biodegradation performance, assist with the selection of inoculants, identify new biodegradative pathways, and eventually to guide efforts in synthetic biology to develop new enzymatic activities (Baek et al., 2007; Yergeau et al., 2012; Uhlik et al., 2013; Dellagnezze et al., 2014; Sierra-Garcia et al., 2014). Having said this, there are still that need to be faced as the technologies mature and, for more information, the reader is referred to the following reviews (Desai et al., 2010; Hazen et al., 2013; Techtmann and Hazen, 2016).

The modern tools of microbial ecology promise to improve our understanding of plant–bacteria synergies and will hopefully lead to better models for designing and deploying effective biological remediation schemes across diverse environmental landscapes.

## Author Contributions

All authors contributed extensively to the work presented in this review. MD and AF provided substantial knowledge on the mechanisms underlying regulation of alkane degradation, whilst JVH and JV contributed with their profound knowledge concerning petroleum microbiology and the role of plant–microbe interactions during phytoremediation, respectively. WS helped in editing the manuscript. PG coordinated and wrote this review.

## Conflict of Interest Statement

The authors declare that the research was conducted in the absence of any commercial or financial relationships that could be construed as a potential conflict of interest.
